# EHMTI-0034. Pharmacological modulation of trigemino-nociceptive stimulation pd06

**DOI:** 10.1186/1129-2377-15-S1-A3

**Published:** 2014-09-18

**Authors:** IL Kröger, A May

**Affiliations:** 1 Department of Systems Neuroscience, University Medical Center Hamburg-Eppendorf, Hamburg, Germany

## Introduction/aims

Using functional resonance imaging (fMRI) with a standardized paradigm for trigemino-nociceptive stimulation [1], we explored the effect of sumatriptan on central pain processing structures compared to saline and acetylsalicylic acid (ASA). Given the mode of action of triptans [2-4], we hypothesized, that differences in BOLD activation between sumatriptan and saline/ASA would be revealed in the trigeminal nuclei.

## Methods

We scanned 21 healthy volunteers for each group (sumatriptan, ASA) at two different time points (within-subject design). Differences in behavioural and imaging data between medication and saline conditions as well as between the medications were investigated. Using a general psychophysiological interaction analysis (gPPI), neuronal coupling between brain structures under saline compared to sumatriptan condition were explored.

This double-blind fMRI study was approved by the local Ethics Committee and all volunteers gave written informed consent prior to fMRI data acquisition.

## Results

Mean pain intensity ratings did not differ between saline and sumatriptan/ASA conditions or between medications. Imaging data reveals increased activation of the trigeminal nuclei (T (18) = 3.59, p< 0.05 FWE corrected) after sumatriptan compared to saline. The same was also true for sumatriptan vs. ASA (T (31) = 2.8, p< 0.05, FWE corrected). The gPPI showed an increased coupling between the trigeminal nuclei and several cortical and subcortical pain related brain areas for the saline condition during painful stimulation.

## Conclusion

The study reveals an increased activation within the trigeminal nuclei under sumatriptan treatment compared to saline. Furthermore, this effect is specific for triptans. As the coupling between the trigeminal nuclei and other pain related brain structures during sumatriptan treatment compared to saline is attenuated, we suggest a weakening effect of sumatriptan on functional brain connectivity during pain.

No conflict of interest.
